# Self-Management Components as Experienced by People with Parkinson's Disease and Their Carers: A Systematic Review and Synthesis of the Qualitative Literature

**DOI:** 10.1155/2020/8857385

**Published:** 2020-12-15

**Authors:** Remco Tuijt, Aylin Tan, Megan Armstrong, Jennifer Pigott, Joy Read, Nathan Davies, Kate Walters, Anette Schrag

**Affiliations:** University College London, London, UK

## Abstract

**Background:**

Self-management strategies are important in healthcare for people with Parkinson's to improve daily living. There is limited evidence on effectiveness in Parkinson's, and the active components of effective self-management strategies are uncertain. This review aims to identify and synthesise the qualitative evidence regarding the experiences of self-management components by people with Parkinson's and their carers.

**Methods:**

MEDLINE, PsycINFO, Embase, Web of Science, and CINAHL were searched from inception to July 8, 2020, for qualitative research concerning self-management for people with Parkinson's. Data were coded and thematically synthesised using NVivo. *Findings*. Of 9547 search results, six papers were included in the final thematic synthesis. The studies reviewed consisted of 147 participants: 104 were people with Parkinson's and 43 were carers. Seven main themes were derived concerning self-management of people with Parkinson's: (1) medication management, (2) physical exercise, (3) self-monitoring techniques, (4) psychological strategies, (5) maintaining independence, (6) encouraging social engagement, and (7) providing knowledge and information. These components should be incorporated as relevant strategies and techniques and should be specific as well as tailored to different stages of the disease. *Discussion*. Self-management programmes for people with Parkinson's should include the seven themes presented as part of this review and pay particular attention to presenting relevant information and skills as they relate to different stages of the disease. Tailoring information and social engagement were two components that required specific attention in order to engage people with Parkinson's effectively.

## 1. Introduction

Parkinson's disease (Parkinson's) is a neurodegenerative disorder that affects an estimated one in 500 people in the United Kingdom [1] and worldwide is the second most common neurodegenerative disorder [2]. A range of motor problems such gait and balance issues, slowness, stiffness and tremor, and nonmotor symptoms can occur, including disturbances of mood and cognitive, autonomic, sensory, and sleep disorders [3]. There is currently no cure for Parkinson's, and medical management needs to balance out the effectiveness of medications and their potential side effects and incorporate nonpharmacological treatments [4]. In order to improve quality of life and symptom management, encouraging the use of self-management strategies for people with Parkinson's has been an increasing focus of recent research [5].

Self-management techniques have been researched with a focus on chronic illnesses such as arthritis or diabetes [6]. Specific components regarding long-term conditions have been derived and developed into the Practical Reviews In Self-Management Support (PRISMS) framework [7]. This 14-part framework identifies specific components of self-management including information, symptom monitoring, and engagement with healthcare professionals. While this provides a comprehensive overview of self-management for long-term conditions, understanding the effectiveness and importance of particular components for people with Parkinson's requires further research [8].

People with Parkinson's have identified some of the components of self-management as a priority [9], in line with expectations of patient-centred care and increased shared decision making [5]. Self-management research with a focus on Parkinson's has highlighted the need for components that support self-efficacy, a sense of coherence, and social support [10]. Due to the diverse needs of people with Parkinson's, it is necessary to provide a wide range of strategies and support, which is reflected in the variability of quantitative outcomes that have been measured in self-management trials [8].

Qualitative research can provide important in-depth information on experiences, expectations, and opinions of participants relevant to the life of a person with Parkinson's [11] and support the development of further self-management programmes. Previous reviews of the qualitative literature have covered general experiences of Parkinson's, but not specifically with regard to self-management [12].

In order to provide recommendations for the development and improvement of self-management programmes and strategies for people with Parkinson's, we aimed to systematically review and synthesise the qualitative evidence regarding the components of self-management as reported by people with Parkinson's and their carers.

## 2. Methods

### 2.1. Search Strategy

This study was designed using reporting guidelines for the synthesis of qualitative research [13]. We initially searched MEDLINE, PsycINFO, Embase, Web of Science, and CINAHL, inception to March 7, 2019, with an updated search on July 8, 2020. Searches were not restricted to any language or date of publication. Target searches combined terms and Medical Subject Headings related to (1) Parkinson's with (2) qualitative research reported in English and (3) self-management experiences using Boolean operators. The full search strategy is available in Appendix A. Inclusion and exclusion criteria are summarised in Table 1. Forward and backward citation searching of relevant articles was conducted as well as grey literature searches using Google Scholar [14].

### 2.2. Study Screening and Data Extraction

One author (AT) extracted and screened all titles and abstracts of the identified studies in accordance with the inclusion criteria, and a second reviewer (RT) conducted a 10% independent interrater reliability check. No discrepancies were found. All full texts were assessed for inclusion by the wider research team.

### 2.3. Methodological Quality

The qualitative version of the Critical Appraisal Skill Programme (CASP) [15] was used to assess methodological quality of the included studies by two authors (RT, MA) independently, with any discrepancies resolved through discussion to find consensus. Quality ratings informed data synthesis and were not used as exclusion criteria.

### 2.4. Thematic Synthesis

Included full text studies were imported into NVivo 11 [16]. The primary qualitative data included within the published papers, the themes reported, and supporting text as well as their related conclusions were coded line by line by two authors (RT and AT) for experiences of self-management components. Following Thomas and Harden's method for thematic synthesis [17], the coded data were analysed for themes related to self-management of Parkinson's, which were inductively derived. For example, “*She likes to do it, but I have to be always by her side because if she falls .... –* Carer, p. 875 [18]” was coded as “carers supporting independence” and “autonomy.” RT and AT together developed initial themes by grouping codes in a hierarchical structure in order to develop thematic synthesis across the included studies. Further synthesis of themes was achieved through discussion with the wider multidisciplinary research team through several meetings, by which “carers supporting independence” and “autonomy” were merged into the theme presented in this paper: “maintaining independence alongside carer support.”

## 3. Findings

**Figure 1:**
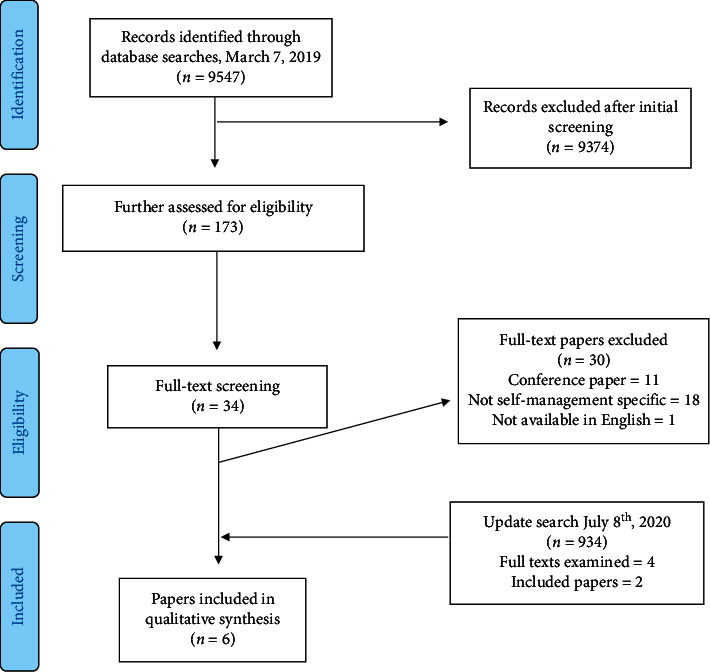
PRISMA flow diagram of included studies.

9547 records were identified in the original database search. After removal of duplicates and screening of abstracts, 34 papers were eligible for full text screening. On full text screening, *n* = 18 were excluded as not focused on self-management of Parkinson's, *n* = 11 were excluded as conference abstracts (no full paper available), and one was not in English with no translation available, leaving four eligible papers from the initial screening. The update search retrieved a further 934 hits, of which four full texts were screened, which identified two additional studies. Please see Figure 1.

### 3.1. Summary of Included Papers

Six papers [18–23] were included in the thematic analysis (see Table 2). In total, the studies consisted of 147 participants: 104 people with Parkinson's and 43 carers. In this review, the use of the term “carer” will refer to family members or friends of the person with Parkinson's. The studies collected data from Canada, New Zealand, Portugal, Sweden, and the United States of America. Disease duration was reported for some of the participants, with varying samples included: one to 13 years living with the disease [19], one to nine years [20, 22], and less than one year to 15 years [18]. In two papers there was no specificity with regard to the participants of the qualitative work [21, 23].

Four papers from three studies explored participant's experiences of a particular self-management programme. This included the Swedish National Parkinson's School (NPS) [19, 20], the Chronic Disease Self-management Programme (CDSMP) [23], and the Living Well With Parkinson's programme (LWWP) [22]. Of the papers that did not evaluate a specific self-management intervention, one paper detailed the codesign of a Parkinson's Care Network [21] and another explored how patients and carers collaborate in self-management [18].

### 3.2. Summary of Included Self-Management Interventions

The NPS was designed from a patient education programme with the aim of providing tools and strategies for people with Parkinson's and their relatives to manage their daily life and promote life satisfaction. The NPS includes seven sessions that include topics like managing stress and presents these in group discussion and through homework assignments [19, 20].

The LWWP programme consisted of six weekly seminars with different topics that lasted one and a half hours, for people with Parkinson's as well as their support person. The main focus of the group was to enable discussion between the participants, in formal settings during the seminar as well as afterwards. Healthcare professionals of different expertise were invited to discuss specific issues related to, for example, research, exercise, nutrition, and the psychological aspects of living with Parkinson's [22].

The CDSMP is a self-management intervention that combines a support group approach with education. As well as sharing relevant information, the intervention includes interactive feedback and problem-solving tasks, as well as sharing personal experiences and strategies. The CDSMP specifically also looks to establish social relationships during the intervention [23].

### 3.3. Methodological Quality of Included Papers

CASP ratings ranged from 8 to 10 out of 10, indicating general high levels of quality in reporting qualitative research. The median of the ratings was 9.5, with three studies not reporting on the relationship between the researcher and the participant and one not clearly stating the aim of the research. Three studies [18, 19, 22] received a 10 rating (see Appendix B for full ratings).

## 4. Synthesis

A thematic analysis of the six papers included in the data synthesis found seven themes around self-management components: (1) medication management, (2) physical exercise, (3) self-monitoring, (4) psychological strategies, (5) maintaining independence, (6) social engagement, and (7) knowledge and information. These themes relate to the self-management strategies people with Parkinson's value, as well as how they approach different challenges in their day-to-day life. Quotations provided as primary data are presented in italics, and the conclusions made by the authors of the papers are quoted in inverted commas.

### 4.1. Medication Management

Medication management was considered across the studies as an important component of self-management. In one of the six studies specifically, people with Parkinson's described different medication management strategies, which varied according to the level of input of their carers and was dependent on the disease stage. Strategies included the use of reminders and alarms, watching the time, and prompting or dispensing of medication by carers [18].

“Reminding about medication can happen in quite different ways, but it seems that patients and carers collaborate in all of them.” – p. 874 [18].

In the other studies, more general comments showed that providing medication information and discussing medication management strategies support and empower people with Parkinson's [19, 20, 22, 23]. Including the person with Parkinson's in medication decisions was also important:

*But that bothers me because eventually the effect of levodopa is to get these other symptoms […]. So that gives me some concern. But we've never really discussed that […]. He just said, ‘I want you to go up to 1,200.' And why I do not know. –* Person with Parkinson's, p. 1360 [21].

Clear communication regarding medication prevented confusion and improved the involvement of the person with Parkinson's in shared decision making and the understanding that medication should be tailored to the individual with regard to their specific symptoms [20].

### 4.2. Physical Exercise

As an important component of self-management, physical exercise was discussed across nearly all studies. Some participants mentioned that targeted exercise, aside from improving general physical and mental well-being, could help with specific symptoms of Parkinson's [20]. Participants reported an increase in motivation for physical activities when in a group setting or when encouraged by a spouse. This was reported both by people with Parkinson's and their carers [19]. Information on the importance of exercise appeared a key aspect of self-management programmes:

*Everybody is always stressing the importance of physical activity and training. And I've been like.. okay... Does it really have an impact at all? Well now we've got proof that it works! I was out walking with her (wife) today and she has never walked so fast! I had a hard time keeping up!* - Carer, p. 3724 [19].

*I exercised before I got this. But I-I've become a real believer in the value of exercise. For delaying the degradation of this disease, so, and I got a lot of that from those programmes from here and other things I've read.* - Person with Parkinson's, p. 92 [23].

Increased knowledge around exercise, including the importance as well as certain techniques or available classes [21, 22], was seen as useful by participants. This was further encouraged by the carers who were seen as important enablers or motivators for the people with Parkinson's [18]. Other strategies to improve motivation for physical exercise included written plans or schedules [19].

### 4.3. Self-Monitoring

For people with Parkinson's, recording symptoms or learning about self-monitoring was an important part of self-management. One of the self-management programmes specifically introduced self-monitoring as a skill to participants [19], but found that people with Parkinson's who were new to the idea found it difficult to grasp and were unsure about its usefulness. However, participants who had several years' experience of living with Parkinson's had developed their own self-monitoring techniques:

*The first 5-6 years of my disease I wrote a diary. I wrote down what I was thinking and feeling and what I could and could no longer do, and coming to conclusions, and I could change the way I was feeling. It was very helpful for me and gave me perspective on my situation.. ..I could make action plans.* – Person with Parkinson's, p. 3723 [19].

It was important to explain the concept beyond “keeping a diary,” as self-monitoring can help an individual better understand and interpret symptoms of their disease and reflect on their self-management behaviour [19]. Along with further knowledge of Parkinson's, self-monitoring could

“[increase] the courage and motivation of [people with Parkinson's] and care partners to engage in self-management activities in everyday life. This promoted self-efficacy and feelings of being in control of their own lives.” – p.6 [20].

One study found that participants self-monitored by using a “tracking log” and reported that this improved healthcare interactions by providing a basis for discussion during a consultation [21]. This was supported by participants of another study who were able to use self-monitoring as way to be more actively involved in decision making [20]. However, healthcare professionals could be dismissive of self-monitoring:

“They have no interest in those things at all. They never talk about it.” – Person with Parkinson's, p. 1359 [21].

The authors concluded that it was important for healthcare professionals to be receptive and encouraging of supporting self-monitoring tools.

### 4.4. Psychological Strategies

The strategies to manage or improve the psychological wellbeing of people with Parkinson's were discussed across studies in differing ways. In some studies, a combination of strategies was included in the self-management programmes, such as relaxation or promoting enjoyable activities [19, 20]. This “positive thinking” was deemed to be well accepted by participants, as well as relaxation activities that encouraged a calmer state of mind which was similarly described as being beneficial:

*If I do things in a relaxed way, without getting nervous, I can do everything. At my speed, I can do it. But if I get nervous, then... –* Person with Parkinson's, p. 874 [18].

In one instance, the self-management programme itself was reported to have boosted the confidence of participants and given them a more positive outlook [22].

An important related topic was acceptance of the condition. Due to the progressive nature of the disease, people with Parkinson's as well as carers had to continually reframe their acceptance over time [18, 22]. Although this was described as a gradual process which could involve denial, sadness, and anger, trying to have a positive mind set and outlook on life was recognised as an important way of trying to live life as well as possible [19].

Accepting the disease as well as accepting the possible future that awaits people with Parkinson's and their carers can bring about stress and anxiety. In order to stop worrying about this, some took an approach that focused on day-to-day life and valuing what they could do and found enjoyable [18, 20]. Another approach included thinking through potential causes of stress and making plans to change the negative thoughts into positive ones [19].

### 4.5. Maintaining Independence alongside Carer Support

This theme relates to the importance of being independent to people with Parkinson's and maintaining that alongside the support they receive on a day-to-day basis from a carer. People with Parkinson's were often motivated to remain active and engaged in order to support their sense of autonomy:

*I don't want to get used to depend [on others]. ... When dressing, for example. Sometimes I have difficulties in putting on my jacket and she [my wife] comes to help, but I don't want [the help]. Because if I get used to it, it is not good.* – Person with Parkinson's, p 876 [18].

This notion of not depending on others for help was shown also in examples where people with Parkinson's indicated they would rather finish a task themselves no matter how many tries it takes before they wanted to ask for help [18]. Nevertheless, the support of carers or family members could facilitate and aid the sense of autonomy of a person with Parkinson's, and accommodations made by a carer could support further engagement and prevent risk:

*She likes to cook but I have to light the gas stove, peel the potatoes, and ... She likes to do it, but I have to be always by her side because if she falls .... –* Carer, p. 875 [18].

The support of the carer could help mitigate the limitations that physical symptoms could have, and people with Parkinson's saw this as beneficial:

*[My care partner and I] recognize skills that maybe I can't do as well as I used to, and instead of being frustrated by something I can't do, just kind of changing roles in my daily life, someone else doing something for me.* – Person with Parkinson's, p. 89 [23].

The support a carer could provide to a person with Parkinson's was multidimensional. They were described as important motivators, and carers themselves expressed a desire to get more knowledge about how to help and to be a support and resource for their partners in managing the disease [19]. This was especially needed in order to support patient-carer dyads where a carer role reversal had taken place and a carer was now navigating the balance of encouraging autonomy of the person with Parkinson's as well as acceptance of the nature of the condition [18, 23].

Carers specifically also had an important supportive role in healthcare interactions, where they could provide their observation of symptoms, and they would often take part in decision making [20]. Collaborating and having open communication with their carers in healthcare settings was recognised by people with Parkinson's as a necessary aspect of managing self-care when asking questions, understanding information, and making decisions [21]. Participants described how this cooperation and understanding was improved when they were provided with specific communication skills and strategies [23]. In order to go over all this, carers in one study suggested that a “relatives only” session would be beneficial as this could be an opportunity for them to talk about problems that they considered may be “too sensitive” to discuss in front of their partner, or in front of the group [19].

### 4.6. Social Engagement

People with Parkinson's often found social engagement valuable as a component of self-management. Group sessions with other individuals who were experiencing similar emotional or physical challenges were highlighted, both within the self-management programmes and for other activities [19, 21–23]. This allowed for peer support from people with lived experience, which encouraged sharing of experiences and information within the groups. Social engagement with people with Parkinson's was important across the differing stages of the disease:

“For those who had lived with [Parkinson's] for many years, the social interaction in a group of peers was considered the most valuable aspect of the NPS, even though the strategies and knowledge taught were not new to them.” – p. 3725 [19].

Social engagement provided an opportunity to share knowledge, namely what other people with Parkinson's had found helpful or what services they had utilised. This was supported by the idea that people who had been in a similar situation would be more honest with one another:

“You get with these people and you sit down and everyone is frank, you know, that's the one thing I really like about it. You don't find people pullin' their punches or anything, you know, they tell you what the problems are and how they've dealt with them, or in some cases, how they haven't dealt with them.” – Carer, p. 91 [23].

Support provided from existing social networks included emotional reliance, a sense of connection and cooperation [23]. Social interaction in general was reported to be important to people with Parkinson's:

“It might not be the activity in itself but the social interaction surrounding the activity. As I told you, we are a couple of friends meeting to prepare good food together. But the cooking itself has actually less importance, it's just getting together... that is what is important!” – Person with Parkinson's, p. 3723 [19].

The effect of specific interventions may differ depending on the existing networks and degree of dependence. Participants in the CDSMP study, which specifically aimed to improve and encourage new social networks, did not feel these had improved as they already had good social networks but also as the intervention needed to be more specific to Parkinson's related social interactions. However, many reported enhanced understanding of and cooperation with existing social networks [23].

### 4.7. Knowledge and Information

The self-management components already mentioned in the themes above were often delivered by providing information in order to increase awareness and understanding. People with Parkinson's felt more empowered to cope with their disease as a result of bringing together information related to different services and resources [22]. Some participants described seeking information as a mechanism to support independence and adapting to life with Parkinson's [19]. It was necessary, however, to combine information alongside specific skills:

“A session teaching self-management skills rather than just giving information on the various aspects of Parkinson's. How to set goals, how and when to change therapy.” - Person with Parkinson's, p. 230 [22].

An important issue that was raised by participants was matching the depth and content of the information about Parkinson's to the stage they were currently in. Specifically, this meant participants felt that, for example, educational content would be more beneficial at an earlier stage of their disease [19]. For those who were already experiencing the symptoms of Parkinson's every day, general information on Parkinson's was unnecessary, and it was not always relevant to the stage of Parkinson's they were in now. This was, however, framed as a great opportunity for those that had been recently diagnosed:

“The two people that had only just been diagnosed, they had everything straight away so they would be…hugely in front of where I was when I was only just diagnosed.” – Person with Parkinson's, p. 230 [22].

Tailoring information and matching it to the stage of the disease was especially important, also with regard to the social engagement theme described previously. One study was carried out in one instance with people with Parkinson's and those who had experienced a mild stroke in a mixed group. The feedback from this stressed the importance of shared peer experience for people with Parkinson's, calling for more participants who had Parkinson's in that group:

“I think if you had more Parkinson's patients, because you can more relate and advise. It's hard for me to envision how to help somebody who's had a stroke and how to say ‘you should – I suggest you do this kind of exercise.'…With Parkinson's, I know what can help. And I just feel like that's a whole different situation than Parkinson's. And it would be more helpful talking to Parkinson's patients one-on-one.” – Person with Parkinson's, p. 92 [23].

## 5. Discussion

The aim of this systematic review was to synthesise the literature regarding the experiences of self-management in people with Parkinson's, exploring the potential components of effective self-management strategies. We identified seven key components that were important in self-management: (1) medication management, (2) physical exercise, (3) self-monitoring, (4) psychological strategies, (5) maintaining independence, (6) social engagement, and (7) knowledge and information. These themes were reported as important components of self-management programmes.

People with Parkinson's found it useful when self-management included specific information about medication and physical exercise, as well as techniques such as self-monitoring and psychological strategies like relaxation techniques. This provided them with ways to manage their Parkinson's across a range of physical or psychological symptoms. People with Parkinson's were usually supported by a carer, and they discussed the importance of finding a balance between the involvement of a carer with the need for people with Parkinson's to be independent and continue managing certain activities themselves. Self-management programmes with a focus on social engagement were well received, although encouraging new social relationships was not always an effective component. Finally, providing participants with knowledge and information was beneficial, especially if this was tailored and combined with specific strategies and skills for the people with Parkinson's.

The themes derived as part of this review regarding the components of self-management for people with Parkinson's overlap with previously established frameworks for long-term conditions [7]. The components of self-management programmes for people with Parkinson's as described in this review were most effective when specific skills and strategies were provided alongside information. This is in line with theories behind self-management interventions that show how the interplay between information, skills, and motivation helps achieve different outcomes [24].

The results of this review, however, highlight the nuance required to make the relevant components suitable for people with Parkinson's. This was apparent with regard to, for example, knowledge and information, as people with Parkinson's noted that information on self-management at an early stage of the disease is highly preferable. In these earlier stages they may also wish to discuss positive actions as opposed to the challenges of the advanced stage of the disease. On the other hand, people who had lived with the disease longer will often have lived experience and may face different challenges, such as the need to continually reframe their acceptance of their condition.

A component where people reported similar issues was social engagement, which has previously been reported as important for people with long-term conditions [25], specifically also for people with Parkinson's [10]. While some participants noted that the social aspect of self-management programmes was generally a useful aspect, there were also people who felt that reliance on social support was indicative of functional decline and thus refrained from developing new relationships [23]. In early stages of the disease, people with Parkinson's may have an established social network that can provide support. Social engagement components of self-management programmes thus need to be carefully tailored to the participant in order to be effective. The findings of this review indicate that for some people developing social engagement specifically for Parkinson's related issues with peers can be useful for providing information, particularly in early stages of the disease. For social and emotional support, a more suitable approach may be to encourage and engage an established social network, such as existing friends and family.

The importance of the support of a carer was apparent throughout various themes, which is in line with previous qualitative work that emphasises how self-management for a person with a long-term condition requires support from family or friends alongside support from professionals and peers [26]. In the context of self-management programmes for Parkinson's, the carer-patient relationship was, however, not clearly explored in the included articles beyond general carer involvement, and research exploring the dynamics of dyadic self-management in Parkinson's is limited [27, 28]. It may be that supporting those navigating changing care-giving relationships is an important component, but this was not covered in detail in the included studies. Additionally, where people with Parkinson's do not have a carer they may need additional support for self-management.

## 6. Strengths and Limitations

This is to our knowledge the first rigorously conducted qualitative systematic review and synthesis of the potential effective components of self-management programmes for Parkinson's. The research team was a multidisciplinary team, which helped provide increased understanding and interpretation. We used clear inclusion and exclusion criteria that were agreed upon by all authors, and multiple authors conducted the full text screening independently. Despite this thorough approach, there are a few limitations to consider.

This review draws on the qualitative work done previously and, as there were only six studies identified, the results should be treated as exploratory to improve further self-management programmes and healthcare delivery. However, the six included studies scored high on the qualitative CASP rating (see Appendix B), providing a source of high quality data for this review, and we identified seven clear components of self-management strategies or programmes with potential for effectiveness as part of this review.

The findings indicate that the level of severity and progression of Parkinson's symptoms should be taken into account with regard to the timing of various components of self-management. Only three studies [19, 20, 23] disclosed the level of disease progression of their participants by using the Hoehn and Yahr scale [29]. Greater specificity and information on the characteristics of participants of such qualitative research studies would strengthen the relevance of the findings and aid implementation.

The definition of self-management can vary in practice, and this may have limited the retrieval of potentially relevant research articles. Our inclusion criteria specified that included papers were to describe an aspect of self-management, in order to focus on discrete components of self-management to inform future strategies and programmes. This may have potentially excluded experiences related to self-management that were not clearly described and highlighted those that were part of specific self-management programmes. We encourage future research to use a clear definition of self-management in order to improve specificity and relevance, especially to clarify distinctions between self-management, self-care, and support [30].

## 7. Implications

This qualitative review of the experiences of people with Parkinson's identified seven key components that are potentially beneficial in the self-management of Parkinson's. Self-management programmes for people with Parkinson's should incorporate information and strategies regarding medication management, physical exercise, self-monitoring, psychological well-being, maintaining independence, and social engagement. Self-management programmes should provide tailored information and strategies that are relevant to the person with Parkinson's. Future self-management research should explore how to support independence alongside developing carer support. Future publications should be encouraged to clearly define self-management and include relevant demographics of their participants, such as the stage of the disease or years living with diagnosis.

## Figures and Tables

**Table 1:** Inclusion and exclusion criteria.

Inclusion criteria	Exclusion criteria
People with idiopathic Parkinson's disease living in the community, or carers of people with Parkinson's disease	Atypical Parkinsonism, life expectancy <6 months, other illnesses, healthy individuals, hospital based (in-patient) populations, or living in a care home
Qualitative research, reported in full text in English	Quantitative research design, survey, closed questionnaire, reviews, protocols, editorials, commentary, conference abstracts, non-English full text papers
Experiences related to self-management such as but not limited to health-related quality-of-life, psychological and physical health outcome, and generalized self-efficacy	Not related to self-management, e.g., diagnosis, service use, end of life care

**Table 2:** Characteristics of included studies.

Author and country	Aim	Participants	Collection	Analysis	Findings
(1) Hellqvist et al., Sweden [19]	To identify and describe experiences valuable for managing daily life after participation in the National Parkinson's School (NPS) self-management intervention	25 people with Parkinson's, 17 relatives	In-depth group discussion	Thematic analysis	Self-management interventions with a cognitive approach introducing techniques aimed at handling the psychological impact of disease in the lives of persons with Parkinson's disease can promote self-care activities and increase life satisfaction. The benefits of the NPS were social connections and support from people in the same situation, as well as improved connection to family, society, and healthcare

(2) Hellqvist et al., Sweden [20]	To study whether people with Parkinson's and care partners implemented the strategies of self-monitoring included in the self-management intervention ‘National Parkinson's School' (NPS) and use them in clinical encounters with healthcare professionals	10 people with Parkinson's, 3 carers	Observations and interviews	Constant comparative analysis	Educational interventions can have an impact on participants' own understanding of Parkinson's disease and the abilities available to handle everyday life. Both participants and care partners valued the intervention. People with Parkinson's disease and care partners reported having adopted the techniques of self-observation, introduced during the NPS, in their lives

(3) Kessler et al., Canada [21]	To develop an integrated approach to care in a tertiary Parkinson's disease clinic by eliciting user experiences of self-management	13 people with Parkinson's, 6 care givers	Interviews	Content analysis	People with Parkinson's and their carers stressed the need for follow-up and coordination of care. They identified the need for provision of relevant information and assistance to access resources and on-going monitoring of their condition. Participants felt better informed and satisfied when professionals practiced open communication and shared decision-making

(4) Mulligan et al., New Zealand [22]	To evaluate an innovative self-management programme for people with Parkinson's disease from the users' perspectives	20 people with Parkinson's, 3 spouses	Interviews	Thematic analysis	A programme of seminars that facilitated identification of strategies for everyday life with Parkinson's was seen as useful in improving individuals with Parkinson's and their support persons' ability to be better at managing life with Parkinson's. While there were benefits to the programme, such as improved knowledge, psychosocial benefits, and new and reinforced strategies, the content received mixed reactions, which could be explained by the participants' individuality, their own journeys, and differing life stages

(5) Nunes and Fitzpatrick, Portugal [18]	To explore how people with Parkinson's and carers collaborate in self-management, and ways in which they adapt everyday arrangements in order to create a meaningful life	9 people with Parkinson's, 8 carers	Interviews and observations	Thematic analysis and grounded theory	People with Parkinson's and their carers engage in numerous collaborations in order to build a life with quality. This joint objective is constantly redefined and renegotiated in face of the current context of Parkinson's disease. Specific self-care activities patients and carers do together to self-manage their condition were explored, that show that collaborations happen independent of the level of autonomy of the person with Parkinson's

(6) Pappa et al., USA [23]	To explore the potential influence of the Stanford Chronic Disease Self-Management Program (CDSMP) on social support in Parkinson's disease	27 people with Parkinson's, 6 care partners	Interviews	Thematic analysis	Participants felt that the workshop does not facilitate relationship building and expressed the desire for explicit encouragement and activities to do so. Although participation did not produce improvements in social support in this study, it did reveal some specific and individualized social support-related benefits to participants and their care partners. Many participants viewed utilization of social support as an indicator of decline and described avoiding the need for social support by adopting behaviours to maintain their independence

## Data Availability

The search strategies used in this systematic review are available in the supplementary data. All data used in this systematic review are from previously reported studies and datasets, which have been cited.

## Supplementary Materials

Supplementary Materials Appendix A: database searches for Medline, Embase, PsycINFO, CINAHL, and Web of Science. Appendix B: critical Appraisal Skills Programme Qualitative Checklist for all included studies.
