# Supplementary material for: Self-Management Components as Experienced by People with Parkinson's Disease and Their Carers: A Systematic Review and Synthesis of the Qualitative Literature
Source: Parkinsons Dis. 2020 Dec 15;2020:8857385. doi: 10.1155/2020/8857385 (PMC7787805; doi:10.1155/2020/8857385)
Supplement: Supplementary Materials — Appendix A: database searches for Medline, Embase, PsycINFO, CINAHL, and Web of Science. Appendix B: critical Appraisal Skills Programme Qualitative Checklist for all included studies. [file 8857385.f1.zip › 8857385.f1/Supplementary data (1).docx]

Supplementary data (Appendix) to: *Self-management components as experienced by people with Parkinson’s disease and their carers: a systematic review and synthesis of the qualitative literature*

**Appendix A**

Medline search

1. exp Parkinson's Disease/ (60940)
2. parkinson*.mp. (117810)
3. 1 or 2 (117810)
4. exp Self Care/ (51343)
5. exp Self-Management/ (980)
6. self care.mp. (40690)
7. self-manag*.mp. (17309)
8. exp Independent Living/ (3936)
9. 4 or 5 or 6 or 7 or 8 (73232)
10. Perception/ (31496)
11. attitude to health/ or health knowledge, attitudes, practice/ (175501)
12. ((experience* or view* or attitude* or thought* or opinion* or perspect* or perceiv* or percept* or feeling* or belie* or value*) adj3 management).mp. (14453)
13. ((experience* or view* or attitude* or thought* or opinion* or perspect* or perceiv* or percept* or feeling* or belie* or value*) adj3 care).mp. (39152)
14. ((experience* or view* or attitude* or thought* or opinion* or perspect* or perceiv* or percept* or feeling* or belie* or value*) adj3 self care).mp. (706)
15. ((experience* or view* or attitude* or thought* or opinion* or perspect* or perceiv* or percept* or feeling* or belie* or value*) adj3 self help).mp. (142)
16. ((experience* or view* or attitude* or thought* or opinion* or perspect* or perceiv* or percept* or feeling* or belie* or value*) adj3 self-management).mp. (722)
17. 10 or 11 or 12 or 13 or 14 or 15 or 16 (250157)
18. exp Qualitative Research/ (44396)
19. ((focus adj2 group) or qualitative or interview* or narrative or ethnograph*).mp. or thematic analysis.ti,ab. (530721)
20. 18 or 19 (530775)
21. 3 and (9 or 17 or 20) (2436)

Embase

1 parkinson*.mp. (196501)

2 exp Parkinson disease/ (142202)

3 1 or 2 (196501)

4 exp self care/ (74544)

5 self-management.mp. (23493)

6 self care.mp. (58076)

7 4 or 5 or 6 (86677)

8 exp attitude to health/ (106000)

9 ((experience* or view* or attitude* or thought* or opinion* or perspect* or perceiv* or percept* or feeling* or belie* or value*) adj3 care).mp. (51169)

10 ((experience* or view* or attitude* or thought* or opinion* or perspect* or perceiv* or percept* or feeling* or belie* or value*) adj3 manag*).mp. (29219)

11 ((experience* or view* or attitude* or thought* or opinion* or perspect* or perceiv* or percept* or feeling* or belie* or value*) adj3 self care).mp. (921)

12 ((experience* or view* or attitude* or thought* or opinion* or perspect* or perceiv* or percept* or feeling* or belie* or value*) adj3 self-manag*).mp. (1033)

13 ((experience* or view* or attitude* or thought* or opinion* or perspect* or perceiv* or percept* or feeling* or belie* or value*) adj3 self-help).mp. (200)

14 8 or 9 or 10 or 11 or 12 or 13 (181560)

15 exp qualitative research/ (61666)

16 ((focus adj2 group) or qualitative or interview* or narrative or ethnograph*).mp. or thematic analysis.ti,ab. (690412)

17 15 or 16 (690514)

18 3 and (7 or 14 or 17) (4562)

PsycINFO

1 exp Parkinson's Disease/ (21117)

2 parkinson*.mp. (33091)

3 1 or 2 (33091)

4 self care.mp. (11459)

5 exp Self-Management/ (6198)

6 self-manag*.mp. (11209)

7 4 or 5 or 6 (21895)

8 ((experience* or view* or attitude* or thought* or opinion* or perspect* or perceiv* or percept* or feeling* or belie* or value*) adj3 care).mp. (17541)

9 ((experience* or view* or attitude* or thought* or opinion* or perspect* or perceiv* or percept* or feeling* or belie* or value*) adj3 manag*).mp. (16582)

10 8 or 9 (33652)

11 ((qualitative or narrative or thematic) adj2 analysis).mp. (30873)

12 (focus adj2 group).mp. (17478)

13 (("semi-structure*" or semistructure* or unstructure* or informal or "in-depth" or indepth or "face-to-face" or structure* or qualitative) adj3 (interview* or discussion* or questionnaire*)).ti,ab,id. (104239)

14 11 or 12 or 13 (138800)

15 3 and (7 or 10 or 14) (477)

CINAHL

S1 (MH “Parkinson Disease”) (17784)

S2 parkinson* (25324)

S3 S1 or S2 (25324)

S4 self management (13314)

S5 (MH “Self Care+”) (42978)

S6 (MH “Community Living”) (12150)

S7 S4 or S5 or S6 (60456)

S8 AB ( ((experience* or view* or attitude* or thought* or opinion* or perspect* or perceiv* or percept* or feeling* or belie* or value*) N3 care) ) (28026)

S9 AB ( ((experience* or view* or attitude* or thought* or opinion* or perspect* or perceiv* or percept* or feeling* or belie* or value*) N3 self care) ) (1210)

S10 AB ( ((experience* or view* or attitude* or thought* or opinion* or perspect* or perceiv* or percept* or feeling* or belie* or value*) N3 management) ) (5788)

S11   AB ( ((experience* or view* or attitude* or thought* or opinion* or perspect* or perceiv* or percept* or feeling* or belie* or value*) N3 self management) ) (772)

S12 S8 or S9 or S10 or S11 (33745)

S13 (MH “Qualitative Studies+”) (124413)

S14 ((focus N3 group) or qualitative or interview* or narrative or ethnograph*) or thematic analysis  (364830)

S15 S13 or S14 (373726)

S16 S3 and (S7 or S12 or S15) (1296)

Web of Science

| # 1 | TS=(parkinson*)  *Indexes=SCI-EXPANDED, SSCI, A&HCI, ESCI Timespan=All years* |
| --- | --- |
| # 2 | TS=("self-care" OR "self-management")  *Indexes=SCI-EXPANDED, SSCI, A&HCI, ESCI Timespan=All years* |
| # 3 | TS=((experience* or view* or attitude* or thought* or opinion* or perspect* or perceiv* or percept* or feeling* or belie* or value*) Near/3 self help)  *Indexes=SCI-EXPANDED, SSCI, A&HCI, ESCI Timespan=All years* |
| # 4 | TS=((experience* or view* or attitude* or thought* or opinion* or perspect* or perceiv* or percept* or feeling* or belie* or value*) Near/3 care)  *Indexes=SCI-EXPANDED, SSCI, A&HCI, ESCI Timespan=All years* |
| # 5 | TS=((experience* or view* or attitude* or thought* or opinion* or perspect* or perceiv* or percept* or feeling* or belie* or value*) Near/3 manag*)  *Indexes=SCI-EXPANDED, SSCI, A&HCI, ESCI Timespan=All years* |
| # 6 | TS=((qualitative OR narrative OR ethnograph* OR thematic) Near/3 analysis)  *Indexes=SCI-EXPANDED, SSCI, A&HCI, ESCI Timespan=All years* |
| # 7 | TS=((semi-structure* OR qualitative) NEAR/3 interview*)  *Indexes=SCI-EXPANDED, SSCI, A&HCI, ESCI Timespan=All years* |
| # 8 | TS=(focus Near/2 group)  *Indexes=SCI-EXPANDED, SSCI, A&HCI, ESCI Timespan=All years* |
| # 9 | #8 OR #7 OR #6  *Indexes=SCI-EXPANDED, SSCI, A&HCI, ESCI Timespan=All years* |
| # 10 | #5 OR #4 OR #3  *Indexes=SCI-EXPANDED, SSCI, A&HCI, ESCI Timespan=All years* |
| # 11 | #1 AND (#2 OR #9 OR #10)  *Indexes=SCI-EXPANDED, SSCI, A&HCI, ESCI Timespan=All years* |

Appendix B

| Studies | Q1 | Q2 | Q3 | Q4 | Q5 | Q6 | Q7 | Q8 | Q9 | Q10 | Total |
| --- | --- | --- | --- | --- | --- | --- | --- | --- | --- | --- | --- |
| 1. Hellqvist et al. (2017) | Y | Y | Y | Y | Y | Y | Y | Y | Y | Y | 10 |
| 1. Hellqvist et al. (2020) | Y | Y | Y | Y | Y | C | Y | Y | Y | Y | 9 |
| 1. Kessler et al. (2019) | N | Y | Y | Y | Y | C | Y | Y | Y | Y | 8 |
| 1. Mulligan et al. (2011) | Y | Y | Y | Y | Y | Y | Y | Y | Y | Y | 10 |
| 1. Nunes & Fitzpatrick (2015) | Y | Y | Y | Y | Y | Y | Y | Y | Y | Y | 10 |
| 1. Pappa et al. (2017) | Y | Y | Y | Y | Y | C | Y | Y | Y | Y | 9 |

1. Was there a clear statement of the aims of the research?
2. Is a qualitative methodology appropriate?
3. Was the research design appropriate to address the aims of the research?
4. Was the recruitment strategy appropriate to the aims of the research?
5. Was the data collected in a way that addressed the research issue?
6. Has the relationship between researcher and participants been adequately considered?
7. Have ethical issues been taken into consideration?
8. Was the data analysis sufficiently rigorous?
9. Is there a clear statement of findings?
10. Is there consideration for the value of the research?

Answer: Y for Yes, N for No, C for Can’t tell

*Critical Appraisal Skills Programme (2018). CASP Qualitative Checklist. [online] Available at:* <https://casp-uk.net/casp-tools-checklists/>
